# miRNA in Circulating Microvesicles as Biomarkers for Age-Related Cognitive Decline

**DOI:** 10.3389/fnagi.2017.00323

**Published:** 2017-10-04

**Authors:** Asha Rani, Andrew O’Shea, Lara Ianov, Ronald A. Cohen, Adam J. Woods, Thomas C. Foster

**Affiliations:** ^1^Department of Neuroscience, McKnight Brain Institute, University of Florida, Gainesville, FL, United States; ^2^Department of Clinical and Health Psychology, University of Florida, Gainesville, FL, United States; ^3^Center for Cognitive Aging and Memory, McKnight Brain Institute, University of Florida, Gainesville, FL, United States; ^4^Genetics and Genomics Program, Genetics Institute, University of Florida, Gainesville, FL, United States

**Keywords:** exosome, microRNA, biomarker, normal aging, Alzheimer’s disease

## Abstract

Community dwelling older individuals from the North Florida region were examined for health status and a comprehensive neuropsychological battery, including the Montreal Cognitive Assessment (MoCA), was performed on each participant. A subpopulation (58 females and 39 males) met the criteria for age (60–89) and no evidence of mild cognitive impairment, with a MoCA score ≥23. Despite the stringent criteria for participation, MoCA scores were negatively correlated within the limited age range. Extracellular microvesicles were isolated from the plasma and samples were found to be positive for the exosome marker CD63, with an enrichment of particles within the size range for exosomes. miRNA was extracted and examined using next generation sequencing with a stringent criterion (average of ≥10 counts per million reads) resulting in 117 miRNA for subsequent analysis. Characterization of expression confirmed pervious work concerning the relative abundance and overall pattern of expression of miRNA in plasma. Correlation analysis indicated that most of the miRNAs (74 miRNAs) were positively correlated with age (*p* <0.01). Multiple regression was employed to identify the relationship of miRNA expression and MoCA score, accounting for age. MoCA scores were negatively correlated with 13 miRNAs. The pattern of expression for cognition-related miRNA did not match that previously described for Alzheimer’s disease. Enrichment analysis was employed to identify miRNA–gene interactions to reveal possible links to brain function.

## Introduction

Normal aging in humans and animal models is associated with changes in specific cognitive processes. Impaired memory, executive function, and processing speed have been well-characterized with advancing age (Alexander et al., 2012; Woods et al., 2013; Febo and Foster, 2016; O’Shea et al., 2016; Nissim et al., 2017; Porges et al., 2017). However, age-related cognitive decline is not uniform, environmental and biological factors including genes, exercise, diet, inflammation, and stress, which are thought to influence the age of onset and the trajectory of cognitive decline (Foster, 2006; Barrientos et al., 2010; Craft et al., 2012; Kumar et al., 2012; Speisman et al., 2013; Cai et al., 2014; Fan et al., 2017). Further complicating the field is the ability to distinguish age-related cognitive decline from diseases that influence cognition (Foster, 2006; Foster et al., 2016).

Neuroimaging, genetics, and circulating biomarkers are being developed to differentiate normal aging from diseases that affect cognition. While genetic markers may suggest susceptibility to disease, these gene markers are not diagnostic. Similarly, more accurate techniques for identifying pathology, such as positron emission computed tomography, are expensive and may miss early diagnosis, which is critical for treatment. Due to the relative ease of collecting blood, blood based biomarkers could provide a simple and relatively inexpensive means for tracking the progression of cognitive decline and effectiveness of treatments, as well as providing information on mechanism for cognitive impairment. Previous work has examined the relationship between cognition and blood biomarkers, based on theories concerning a role for lipids and cholesterol, oxidative stress, hormones, and inflammation in promoting disease and senescent physiology (Foster, 2006). Recent research suggests that non-coding RNAs found in the circulation can act as biomarkers for diseases of aging including cancer, cardiovascular and neurodegenerative disease (Sheinerman and Umansky, 2013; Schwarzenbach et al., 2014; Mushtaq et al., 2016; Schulte et al., 2016).

MicroRNAs (miRNAs) are small, phylogenetically conserved, 18–25 base pair sections of RNA that influences biological processes through the post-transcriptional regulation of RNA. miRNA acts as a template for target mRNA, binding to the 3′ untranslated region (UTR) of mRNA to silence genes by inhibiting translation and initiating mRNA degradation of the target mRNA. Some miRNAs are ubiquitously expressed in order to regulate fundamental metabolic pathways and variability in expression is influenced by ongoing physiology, including aging. In other cases, miRNAs are preferentially expressed in specific tissues or during different times of development and maturation (Landgraf et al., 2007; Shao et al., 2010; Fehlmann et al., 2016). Finally, examination of the brain suggests that intracellular miRNA signaling influences neural circuits, including those associated with psychiatric diseases (Impey et al., 2010; Serafini et al., 2014; Jovasevic et al., 2015; Rajman et al., 2017).

Within the circulation, miRNAs can be found attached to proteins or in extracellular vesicles, small (50 nm to 1 μm) vesicles of endocytic origin that are released from cells into the extracellular environment. Some (e.g., exosomes) are able to cross membranes (e.g., blood–brain barrier) and can be detected in bodily fluids including serum, urine, and saliva. In this way, microvesicles can provide intercellular and inter-organ communication by delivery of miRNAs to influence transcription and altering genetic processes. Indeed, studies suggest that circulating levels of miRNAs in plasma (Kumar et al., 2013) or in exosomes (Cheng et al., 2015; Lugli et al., 2015) may be able to identify Alzheimer’s disease. The current study employs the datasets from previous studies examining the relationship of brain structural and cognitive function in older adults (O’Shea et al., 2016; Nissim et al., 2017). We now include additional analysis of the expression of miRNAs, isolated from plasma enriched for microvesicles, and relate the expression to cognition in advanced age.

## Materials and Methods

### Participants

The study was approved by the Ethics Review Committee on Human Research of the University of Florida (Gainesville, FL, United States) and written informed consent was obtained from all participants. The participants were selected from previous studies (O’Shea et al., 2016; Nissim et al., 2017) in which healthy community dwelling older individuals were recruited from Gainesville and the North Florida region. A thorough medical history questionnaire for each participant provided detailed information on health status, medication status, and a comprehensive neuropsychological battery was performed on each participant (O’Shea et al., 2016; Nissim et al., 2017). No participants in this sample were clinically indicated to have mild cognitive impairment (MCI) or other age-related brain disorders. The Montreal Cognitive Assessment (MoCA) was given to assess general cognitive ability as well as rule out possible MCI (Nasreddine et al., 2005). From this group, we selected those between the ages of 60–89, with a MoCA ≥23. The 97 participants met the criteria for inclusion in this study, with 58 females and 39 males.

### Sample Collection and Microvesicle Characterization

The plasma samples were collected into EDTA Tubes- Plasma (Cat# 367863). The tubes were inverted five times, stored on ice and processed within 30 min of blood draw. The samples were centrifuged at 1600 × *g* for 15 min at 4°C and the isolated plasma samples were stored at -80°C until RNA isolation. The plasma was filtered (0.22 μm filter; Millipore, Billicera, MA, United States) to remove cellular material, including thrombocyte fragments. Microvesicles were isolated using the exoEasy Maxi and exoRNeasy kit (Qiagen).

For a subset of samples, the size distribution and concentration of the microvesicles were determined by University of Florida Interdisciplinary Center for Biotechnology Research using the NanoSight 300 Instrument (Malvern Instruments), according to the manufacture instruction. In addition, morphological assessment of microvesicles was determined by University of Florida Interdisciplinary Center for Biotechnology Research using the transmission electron Microscopy (TEM). A glow discharged carbon coated Formvar copper 400 mesh grid, was floated onto 10 microliter aliquots of re-suspended microvesicle pellet and incubated for 5 min. Excess solution was drawn off with filter paper and the grids were floated on 1% aqueous uranyl acetate for 30 s. Stain was removed with filter paper, air dried and examined using FEI Tecnai G2 Spirit Twin TEM (FEI Corp., Hillsboro, OR, United States) and digital images were acquired with Gatan UltraScan 2k × 2k camera and Digital Micrograph software (Gatan Inc., Pleasanton, CA, United States).

### RNA Isolation

RNA was isolated using exoRNeasy Serum/Plasma Maxi kit (Cat# 77064, Qiagen) according to the manufacturer’s instructions with the final elution volume of 12 μl. The quantity and quality of the RNA were determined by University of Florida Interdisciplinary Center for Biotechnology Research using the Agilent RNA 6000 Pico Kit to determine the concentration of total RNA, and a Small RNA Kit Chip was used to measure the concentration of exosomal micro RNA (miRNA) on the Agilent Bioanalyzer instrument (Agilent Technologies). Total RNA samples contained a range of 49–90% miRNA.

### Small RNA Library Preparation

Sequencing libraries were constructed using ∼2 ng of total exosomal RNA with the library preparation kit Ion Total RNA-Seq kit v2 (Thermo Fisher, Cat# 4475936). Each library was barcoded with Ion Xpress RNA Seq-Barcode 01-16 Kit (Thermo Fisher, Cat# 4475485) to enable multiplex sequencing. The concentration of the libraries was quantified by the Qubit dsDNA HS Assay (Thermo Fisher, Cat# Q32851). In addition, the size distribution and molar concentration was determined with the High Sensitivity D1000 Screen Tape Kit (5067–5584) on 2200 TapeStation system (Agilent Technologies, Cat# G2964A) according to the manufacture’s protocol.

### Sequencing, Data Acquisition, and Bioinformatics

Templates were prepared with 25 μl of the pooled libraries at a final concentration of 50 pM using Ion Chef instrument (Thermo Fisher) and then sequenced in the Ion Proton System (Thermo Fisher). FASTQ files were extracted from Ion Torrent server and uploaded to the Partek Flow (Partek Inc., St. Louis, MO, United States) servers for bioinformatics analysis. On average, each sample contained 13.8 million reads of 32 base pair (bp) length. Reads were trimmed based on size such that reads below 15 bp and reads above 35 bp were discarded. Following trimming, reads were aligned using Bowtie (version 1.0.0) against the human genome reference (hg38) followed by a post-alignment quality check to assess the performance of the alignment. Gene annotation was completed with the miRBase mature miRNAs model (release version 21) and normalization was performed on total counts. In order to consider genes that are not present in miRBase, gene annotation was also done using hg38-Ensembl Transcripts (release version 85) followed by total count normalization. (Gene Expression Omnibus accession number: The data for this study has been uploaded to the Gene Expression Omnibus under the accession number GSE97644.

To study functionally related genes and their relationship, a biological interpretation was performed by gene ontology (GO) enrichment analysis using DIANA tool web-based software using mirPath (v.3) for miRNA pathway analysis and TarBase (v7.0) (Vlachos et al., 2015) was employed to identify miRNA–gene interactions. The statistical cutoff for GO analysis was based on corrected *p*-values with the Benjamini–Hochberg’s False Discovery Rate (FDR) *p* < 0.05.

### Statistical Analysis

For statistical analysis, miRNA counts were first log-transformed. Differences associated with sex were determined using analyses of variance (ANOVAs). Pearson’s regression analysis was used to examine correlations associated with age and miRNA expression. Due to the correspondence of age and cognitive function, multiple regression was employed to determine correlation of miRNA with MoCA score after adjusting for age.

## Results

**FIGURE 1:**
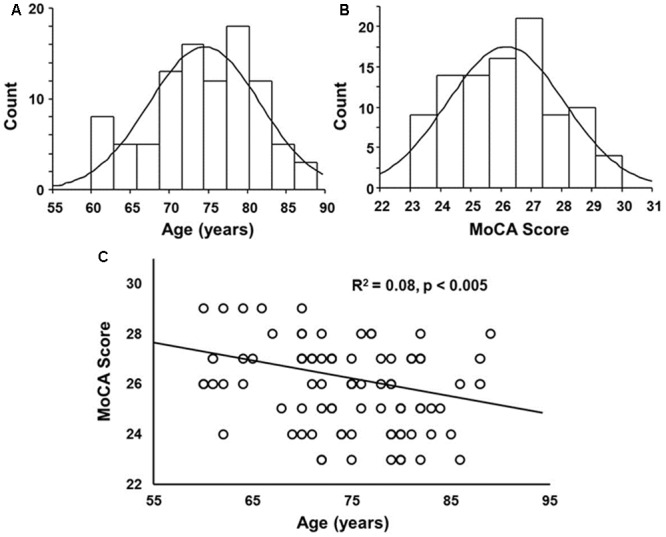
Histograms of the distribution of **(A)** ages and **(B)** Montreal Cognitive Assessment (MoCA) scores overlaid with a normal distribution curve based on the mean and SD. **(C)** A correlation was observed between age and MoCA score, such that the MoCA score decreased with increasing age.

Cognitive testing and plasma were collected from a total of 134 participants, age range 44–102 years. From this population, five were removed due to a history suggesting possible brain disorders due to stroke, concussion, or epilepsy, 22 were removed due to a MoCA score less than 23 (MoCA score range 18–22), and 10 were outside the specified age range of 60–89 years, with one person age 92 and 9 individuals 44–59 years. The remaining 97 participants that met the criteria for inclusion in this study included 58 females and 39 males. The distribution of ages and MoCA scores was approximately normal (**Figure 1**). No age difference was observed between females (75.4 ± 1.0 years mean ± SEM) and males (mean 73.0 ± 1.0 years). While we cannot rule out that some participants may have been pre-symptomatic for MCI or Alzheimer’s disease, the MoCA scores were negatively correlated with age (*R*^2^ = 0.08, *p* < 0.005) (**Figure 1C**), suggesting that this population exhibited an age-related cognitive decline.

### Extracellular Microvesicle Characterization

**FIGURE 2:**
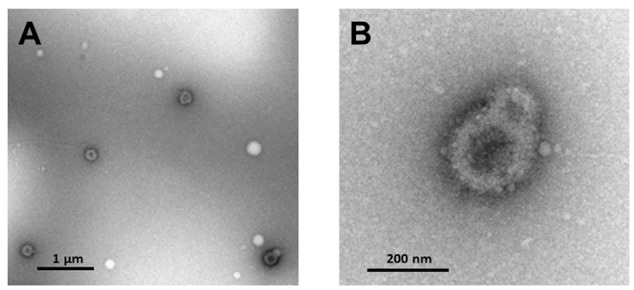
Transmission electron microscopy of **(A)** extracellular microvesicles isolated from plasma and negatively stained with 1% uranyl acetate. **(B)** Enhanced magnification of microvesicles shown in lower right corner of **(A)**.

Characterization of extracellular microvesicle markers was performed for five samples. Elisa assays indicated that all samples were positive for the exosome marker CD63 (OD_450_ 4.08^-1^ ± 0.24, mean ± SD) (Peterson et al., 2015). NanoSight analysis indicated an average vesicle size of 188.82 ± 22.9 nm (mean ± SD), average mode 152.18 ± 34.7 (mean ± SD), and the percent of particles that were <200 nm averaged 65%. Electron microscopy confirmed the recovery of small vesicles with an expected size range from 50 to 200 nm (**Figure 2**).

### miRNA

For expression of miRNA, a cut-off was set such that expression had to average at least 10 across the 97 participants. This filtering resulted in data for 117 miRNAs for analysis. Similar to previous reports (Hunter et al., 2008; Mooney et al., 2015), we observed that mir-223-3p exhibited the highest level expression and relatively high level expression (>300 average counts) was observed for mir-191-5p, mir-126-3p, mir-126-5p, mir-484, and mir-26a-5p (**Table 1**).

**Table 1:** Highly expressed miRNA.

miRNA	Average expression
hsa-miR-223-3p	8760.771
hsa-miR-451a	2372.06
hsa-miR-191-5p	1548.376
hsa-miR-126-3p	1174.333
hsa-miR-126-5p	1021.754
hsa-miR-103a-3p	824.9511
hsa-miR-23a-3p	804.3156
hsa-miR-26a-5p	796.5815
hsa-miR-19b-3p	717.237
hsa-miR-150-5p	697.2409
hsa-miR-484	654.5321
hsa-let-7a-5p	557.691
hsa-miR-185-5p	436.1216
hsa-miR-320a	361.9573
hsa-miR-22-3p	349.7947
hsa-let-7b-5p	325.3197
hsa-let-7g-5p	302.027

*For all tables, expression represents averaged counts.*

An examination of differential expression between the males and females indicated no difference in miRNA expression. Previous work suggests expression of circulating miRNAs increases with increasing age (Freedman et al., 2016). Therefore, Pearson’s regression analysis with a cut-off of *r* = ±0.263 (*p* < 0.01) was employed to examine correlations of miRNA expression with age. The results revealed 74 miRNAs that were positively correlated with age (**Table 2**), confirming that most plasma miRNA that changes with age, exhibit increased expression with advancing age (Freedman et al., 2016). Interestingly, several miRNA that are consistently reported to decrease in blood, plasma, and serum of Alzheimer’s patients (hsa-let-7g-5p, hsa-let-7e-5p, and hsa-miR-103a-3p) (Kumar et al., 2013; Leidinger et al., 2013; Tan et al., 2014; Satoh et al., 2015; Nagaraj et al., 2017), were positively correlated with age.

**Table 2:** miRNA correlated with age.

miRNA	*r*-Value	Average expression	miRNA	*r*-Value	Average expression
hsa-miR-423-5p	0.40	192.63	hsa-miR-126-5p	0.30	1021.75
hsa-miR-145-5p	0.37	80.97	hsa-miR-134-5p	0.30	19.46
hsa-miR-425-5p	0.37	240.80	hsa-miR-19a-3p	0.30	179.28
hsa-miR-22-5p	0.36	10.33	hsa-miR-339-3p	0.30	13.74
hsa-miR-140-5p	0.35	11.19	hsa-miR-29a-3p	0.30	67.12
hsa-miR-376a-3p	0.35	25.80	hsa-miR-199a-5p	0.30	57.71
hsa-miR-185-5p	0.34	436.12	hsa-miR-425-3p	0.30	14.47
hsa-miR-23b-5p	0.34	66.37	hsa-miR-424-5p	0.30	16.52
hsa-miR-23a-3p	0.34	804.32	hsa-miR-660-5p	0.30	10.43
hsa-miR-652-3p	0.34	22.15	hsa-miR-15a-5p	0.30	116.76
hsa-miR-25-3p	0.33	35.00	hsa-miR-339-5p	0.29	37.97
hsa-miR-128-3p	0.33	25.74	hsa-miR-99b-5p	0.29	47.06
hsa-miR-30d-5p	0.33	119.15	hsa-miR-136-5p	0.29	11.01
hsa-miR-485-3p	0.33	27.98	hsa-miR-27a-3p	0.29	105.93
hsa-miR-22-3p	0.33	325.32	hsa-miR-19b-3p	0.29	697.24
hsa-miR-221-3p	0.33	129.56	hsa-miR-625-3p	0.29	26.04
hsa-miR-382-5p	0.33	19.56	hsa-miR-106b-5p	0.29	29.31
hsa-miR-484	0.33	654.53	hsa-miR-103a-3p	0.29	824.95
hsa-miR-29c-3p	0.32	26.14	hsa-miR-199a-3p	0.29	78.57
hsa-miR-21-5p	0.32	190.64	hsa-miR-18a-5p	0.29	258.39
hsa-miR-92a-3p	0.32	203.81	hsa-miR-17-3p	0.29	20.08
hsa-miR-33a-5p	0.32	25.59	hsa-miR-186-5p	0.29	11.36
hsa-miR-421	0.32	11.50	hsa-miR-148a-3p	0.29	16.18
hsa-miR-146a-5p	0.32	152.48	hsa-miR-29b-3p	0.29	30.96
hsa-miR-376c-3p	0.32	227.40	hsa-miR-197-3p	0.29	123.44
hsa-let-7d-3p	0.32	32.72	hsa-let-7e-5p	0.29	34.63
hsa-miR-24-3p	0.31	246.04	hsa-miR-20a-5p	0.29	125.10
hsa-miR-664a-3p	0.31	10.75	hsa-miR-423-3p	0.28	255.35
hsa-miR-28-3p	0.31	20.59	hsa-miR-27b-3p	0.28	29.59
hsa-miR-766-3p	0.31	44.77	hsa-miR-199b-3p	0.28	39.08
hsa-miR-28-5p	0.31	20.26	hsa-miR-223-3p	0.28	8760.77
hsa-miR-590-5p	0.31	78.01	hsa-miR-378a-3p	0.28	22.60
hsa-miR-324-5p	0.31	18.93	hsa-let-7i-5p	0.28	94.64
hsa-miR-584-5p	0.31	69.43	hsa-miR-15b-3p	0.27	11.55
hsa-miR-1307-3p	0.30	10.92	hsa-miR-186-5p	0.27	11.36
hsa-miR-93-5p	0.30	141.86	hsa-miR-574-3p	0.27	39.23
hsa-miR-361-5p	0.30	27.69	hsa-let-7g-5p	0.26	302.03

**FIGURE 3:**
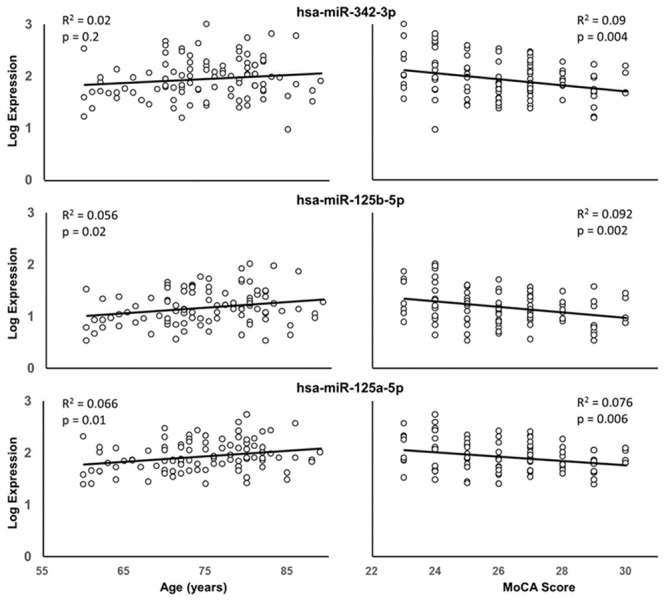
Correlation of hsa-miR-342-3p **(top)**, hsa-miR-125b-5p **(middle)**, and hsa-miR-125a-5p **(bottom)** expression with age **(left)** or MoCA scores **(right)**. The *R*^2^ and *p*-values for the simple regressions are provided.

**FIGURE 4:**
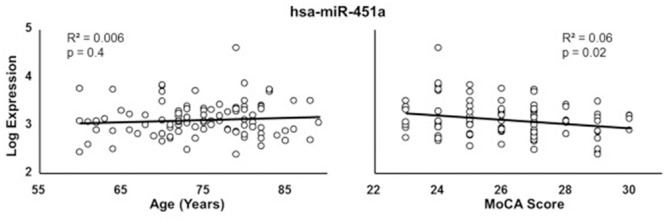
Correlation of hsa-miR-451a with age **(left)** or MoCA scores **(right)**.

Due to the correspondence of age with miRNA expression and cognitive function, multiple regression was performed to examine the relationship of miRNA expression to MoCA scores, accounting for the influence of age. The analysis indicated that 13 miRNA exhibited a significant correlation with MoCA scores (**Table 3**) and 16 exhibited a trend (*p* > 0.05 < 0.1). In all cases, the correlations were negative such that increased miRNA expression was associated with decreased MoCA scores. Interestingly, three of the cognition related miRNA from **Table 3** exhibit relatively selective expression in the brain (hsa-miR-342-3p, hsa-miR-125b-5p, hsa-miR-125a-5p) (Hinske et al., 2014). Moreover, hsa-miR-342-3p and hsa-miR-125b-5p exhibited the strongest correlation with MoCA scores (**Table 3**). These three miRNAs exhibited relatively poor correlation with age (**Figure 3**). **Figure 4** illustrates the age and MoCA score correlations for has-miR-451a-3p, which exhibited the highest expression (**Table 3**), was correlated with cognition, and did not exhibit a correlation with age.

**Table 3:** MoCA score multiple regression analysis.

	Coefficients			
miRNA	miRNA	Age	*R*^2^	Expression
hsa-miR-342-3p	–1.26^∗∗^	–0.069^∗^	0.15	149.03
hsa-miR-125b-5p	–1.42^∗^	–0.063^∗^	0.14	21.73
hsa-miR-10a-5p	–1.39^∗^	–0.065^∗^	0.14	79.04
hsa-miR-140-3p	–1.74^∗^	–0.065^∗^	0.14	14.23
hsa-miR-451a	–1.14^∗^	–0.074^∗∗^	0.13	2372.06
hsa-miR-99a-5p	–1.03^∗^	–0.067^∗^	0.13	52.26
hsa-miR-23b-3p	–1.37^∗^	–0.066^∗^	0.13	64.87
hsa-miR-10b-5p	–1^∗^	–0.069^∗^	0.13	84.84
hsa-miR-125a-5p	–1.41^∗^	–0.064^∗^	0.13	114.18
hsa-miR-186-5p	–1.28^∗^	–0.063^∗^	0.13	11.59
hsa-miR-378a-3p	–1.74^∗^	–0.063^∗^	0.13	22.60
hsa-miR-26b-5p	–1.14^∗^	–0.065^∗^	0.13	49.22
hsa-miR-30c-5p	–1.01^∗^	–0.065^∗^	0.12	15.67

*Asterisks indicate a significant contribution ^∗^*p* < 0.05, ^∗∗^*p* < 0.01.*

To study functionally related genes and their relationship, a biological interpretation was performed by GO enrichment analysis using DIANA tool web-based software to identify miRNA–gene interactions. To understand possible mechanisms through which miRNA could influence the brain, miRNA that correlated with MoCA scores were submitted to DIANA for miRNA pathway analysis. Combining the three brain selective miRNA (hsa-miR-342-3p, hsa-miR-125b-5p, hsa-miR-125a-5p) cluster enrichment indicated the top three pathways were associated with fatty acid biosynthesis (3 genes, *p* = 1.9^-16^), hippo signaling (38 genes, *p* = 1.8^-9^), and protein processing in the endoplasmic reticulum (43 genes, *p* = 7.5^-6^). Brain specific pathways included neurotrophin signaling (27 genes, *p* = 0.016). For the highest expressing miRNA, hsa-miR-451a (**Table 3**), DIANA analysis indicated brain related cluster enrichment for Parkinson’s disease (2 genes, *p* = 1.0^-5^) and glioma (3 genes, *p* = 0.02), and clusters for signaling pathways related to aging, including mTOR signaling (6 genes, *p* = 0.002) and AMPK signaling (6 genes, *p* = 0.027). When all 13 miRNAs that correlated with MoCA scores were loaded into DIANA, the results indicate several pathways associated with brain function including prion disease (20 genes, 11 miRNA, *p* = 1.0^-6^), glioma (44 genes, 13 miRNA, *p* = 2.9^-6^), Huntington’s disease (102 genes, 11 miRNA, *p* = 0.015), and axon guidance (71 genes, 12 miRNA, *p* = 0.019).

## Discussion

The results provide evidence that miRNA, from extracellular microvesicle enriched plasma samples, correlates with cognitive function in healthy elderly individuals. In discussing these results, there are several caveats that need to be considered. Exosomes have the potential to cross the blood–brain barrier and could provide a marker of brain health (Alvarez-Erviti et al., 2011). Alternatively, exosomes in the plasma could cross into the brain to deliver their cargo and influence brain function. Thus, it is important to consider the enrichment of exosomes. Elisa assays indicated that the samples were positive for the exosome marker CD63 indicating that the samples are enriched in exosomes. The enrichment of exosomes was supported by examination of microvesicle size, with ∼65% of particles in the range of exosomes. Exosomes are classically defined as 50–150 nm in diameter, although larger extracellular vesicles (>200 nm) have been described (Kowal et al., 2016). The 150 nm limit may represent a bias due to isolation techniques, and ignores the possible functional impact of increased volume of larger vesicles that have been described (van der Pol et al., 2012; Kowal et al., 2016). Regardless, it appears that the samples were enriched in exosomes.

A second consideration concerns the stringent criteria for the population of participants. Participants were screened in an attempt to exclude those with dementia or Alzheimer’s disease. Interestingly, hsa-let-7g-5p, which is commonly found to decrease in plasma, serum, and blood from Alzheimer’s patients (Kumar et al., 2013; Tan et al., 2014; Satoh et al., 2015), was observed to increase with age in our plasma samples and in previous studies using a wider age range (Freedman et al., 2016). Other miRNAs that were increased with age in our study and the Freedman study, and yet have been reported to decrease in blood or plasma from Alzheimer’s patients, include hsa-let-7e-5p and hsa-miR-103a-3p (Kumar et al., 2013; Leidinger et al., 2013; Satoh et al., 2015; Nagaraj et al., 2017). In all cases, multiple regression indicated no correlation of these miRNA with the MoCA score when age was taken into consideration. The stringent criteria and the absence of suspected miRNA markers of Alzheimer’s disease increases the confidence that these individuals did not have a neurodegenerative disease; although it is possible that they were pre-symptomatic. On the other hand, our results indicating that these miRNAs increase with age, emphasizing the importance of considering age when investigating biomarkers of disease. It has been suggested that the inability to reproduce expression differences may result from age differences across cohorts (Satoh et al., 2015; Cosin-Tomas et al., 2017; Nagaraj et al., 2017), and for studies that found decreased miRNA expression associated with Alzheimer’s disease, the disease and control groups were age-matched.

Mild cognitive impairment is considered a transitional state between normal aging and Alzheimer’s disease and previous research suggests a threshold cutoff MoCA score of 19–23 for designating MCI (Luis et al., 2009; Dong et al., 2012; Larner, 2012; Freitas et al., 2013). Previous studies have reported that specific miRNAs increase in plasma or serum in MCI patients (Sheinerman et al., 2013; Dong et al., 2015). In many cases, the previously reported miRNA exhibited expression levels below our cutoff and were not considered. The stringency for expression reduces the likelihood of type I error and increases the confidence in those miRNA that were correlated with age or MoCA scores. However, the stringency makes it likely that we missed low expressing miRNA that correlate with cognitive function. Indeed, considering that all cognition-related miRNAs exhibited increased expression associated with a decline in MoCA score, it is likely that we missed miRNA that exhibited low expression, particularly in cognitively intact individuals. In the case of miRNA that did satisfy our stringent cutoff, and have been reported to increase in MCI patients, expression of hsa-miR-128-3p, hsa-miR-134-5p, hsa-miR-382-5p, hsa-miR-146a-5p, and hsa-miR-93-5p was observed to increase with age and was not correlated with the MoCA score. We cannot rule out that individuals were pre-symptomatic for MCI. Thus, it will be important for future studies to track cognitive changes as well as miRNA markers over time to determine if miRNA are predictive of decline associated with normal aging and disease.

In comparing the pattern of miRNA expression to previous work, it is important to recognize that much of the previous work has examined miRNA directly from plasma or serum, which includes exosomal and protein-bound miRNAs. It is expected that miRNA expression of plasma and exosomal enriched samples will be similar for many miRNAs; however, expression of some miRNAs may differ (Freedman et al., 2016). Our results confirm a high level of expression for mir-223-3p, mir-451a, mir-191-5p, mir-126-3p, mir-126-5p, mir-484, and mir-26a-5p, which are enriched in plasma exosomes (Hunter et al., 2008; Pritchard et al., 2012; Cheng et al., 2014; Mooney et al., 2015). Second, previous work indicates that chronological age provides a strong influence on expression of plasma miRNA. Indeed, we found over half the exosomal miRNAs examined at *p* < 0.01, exhibited increased expression with age (false discovery rate *p* < 0.016). The robustness of these 74 age-related miRNAs is emphasized by the fact that a well powered study reported 51 of these miRNAs increased in plasma across a broader age range (Freedman et al., 2016). Together, the results indicate that miRNA provide a good marker for chronological age. Together, the results emphasize that age is a major risk factor for Alzheimer’s disease and MCI. Thus, it should not be surprising that biological markers of chronological age provide good predictors of diseases. As such, age should be taken into account when attempting to link biological markers to age-related diseases.

Due to the correlation of age with measures of cognitive function and miRNA expression, multiple regressions were employed to examine the relationship between MoCA scores and miRNA expression, in order to account for age effects. Our analysis point to several miRNAs that may be good predictors of cognitive function in elderly individuals. Exosomes have the potential to cross the blood–brain barrier (Alvarez-Erviti et al., 2011). Thus, highly expressed miRNAs coming from white (mir-223-3p, mir-191-5p, mir-150-5p, mir-26a-5p, mir-19b-3p) or red (mir-451a) red blood cells (RBCs) (Hunter et al., 2008; Pritchard et al., 2012) could influence brain function. In the current study, we observed an increase in hsa-mir-19b-3p with age; however, this miRNA was not correlated with cognition. In contrast, MoCA scores were correlated with hsa-mir-451a. One possible confound is that hemolysis can increase the level of RBC enriched miRNA, hsa-mir-451a and hsa-mir-16-5p, in plasma and serum (Blondal et al., 2013; Kirschner et al., 2013), which could have contaminated our microvesicle enriched samples. A number of factors can influence the fragility of RBCs. However, it is important to note that the fragility of RBCs declines over the course of aging (Penha-Silva et al., 2007; de Freitas et al., 2014). Furthermore, when variability in cognition associated with age was taken into account, expression of hsa-mir-451a, but not hsa-mir-16-5p, was correlated with the MoCA scores. On the other hand, RBCs are not the only source of hsa-mir-451a and an increase in release of hsa-mir-451a containing microvesicles is associated with disease in other cell types as well as senescence of platelets (Ji et al., 2014; Dickman et al., 2017; Pienimaeki-Roemer et al., 2017; Takikawa et al., 2017). The manner in which hsa-mir-451a could influence cognition is unknown; however, an increase in plasma hsa-mir-451a has been reported to be associated with vascular dementia (Prabhakar et al., 2017) and increased expression in the brain may alter synaptic function (Mor et al., 2015).

The MoCA scores were also correlated with several miRNAs that are enriched in the brain (Hinske et al., 2014). An increase in brain specific miRNA in the plasma could result from increased leakiness of the blood–brain barrier, increased expression of specific miRNA, increased release of brain derived microvesicless or a combination. If an increase in plasma levels was due to increased leakiness of the blood–brain barrier, we might expect to have seen a whole host of brain specific miRNAs increase in the plasma of impaired individuals. This does not seem to be the case since other brain selective miRNAs (e.g., hsa-miR-320b, hsa-miR-328-3p, hsa-miR-744-5p) passed our expression criterion and did not correlate with MoCA score. On the other hand, an increase in brain selective miRNAs in the plasma may represent increased release of specific miRNA due to aberrant neural activity, damage, or disease (Lachenal et al., 2011; van der Vos et al., 2011; Wang et al., 2011; Serafini et al., 2014; Harrison et al., 2016).

## Conclusion

In the current study, we describe miRNAs associated with extracellular microvesicles from plasma as possible biomarkers of cognitive decline during aging. A decrease in MoCA score was associated with increased expression of several miRNAs. The rise in expression of brain selective miRNA could signify conditions in the brain, such as aberrant neural activity, damage, or disease, that result in increased synthesis or release from the brain and a decline in function. In addition, it is possible that highly expressed miRNA are delivered to the brain from the circulation, to influence brain function. The miRNA biomarkers from plasma microvesicle exhibited an expression profile, which was different from that previously described for Alzheimer’s disease, suggesting that these biomarkers may be specific to cognitive decline in normal aging. Alternatively, these miRNAs may be related to a pre-symptomatic stage of disease.

## Author Contributions

AR performed experiments. AO, AW, and RC collected behavior data. LI helped in analysis. TF designed experiments, analyzed data, constructed figures, and wrote manuscript.

## Conflict of Interest Statement

The authors declare that the research was conducted in the absence of any commercial or financial relationships that could be construed as a potential conflict of interest.
